# Courtship behavior and identification of a sex pheromone in *Ibalia leucospoides* (Hymenoptera: Ibaliidae), a larval parasitoid of *Sirex noctilio* (Hymenoptera: Siricidae)

**DOI:** 10.7717/peerj.12266

**Published:** 2021-11-04

**Authors:** Hajar Faal, Peter J. Silk, Peter D. Mayo, Stephen A. Teale

**Affiliations:** 1 Department of Environmental and Forest Biology, State University of New York-Environmental Science and Forestry, Syracuse, NY, USA; 2 Forest Pest Methods Laboratory (Otis Laboratory), USDA-APHIS-PPQ-CPHST, Buzzards Bay, MA, United States of America; 3 Natural Resources Canada, Atlantic Forestry Centre, Fredericton, New Brunswick, Canada

**Keywords:** Courtship behavior, Sex pheromone, Cuticular hydrocarbon, Parasitoid, Ibaliidae

## Abstract

**Background:**

*Ibalia leucospoides* (Hymenoptera: Ibaliidae) is a larval parasitoid that has been widely introduced as a biological control agent for the invasive woodwasp*,Sirex noctilio* (Hymenoptera: Siricidae) in the Southern Hemisphere. In this study, the courtship behavior and identificaion of sex pheromones are described for *I. leucospoides* under laboratory conditions.

**Methods:**

For courtship behavior, both sexes were observed in a wire mesh observation cylinder (75 cm length ×10 cm diameter) for 15 minutes. The female body washes were analyzed using Gas Chromatography- Electroantennographic Detection (GC-EAD). Then the EAD-active compounds were tentatively identified using GC-Mass Spectrometry (GC-MS) and examined in olfactometer assays.

**Results:**

The courtship behavior included rhythmic lateral movements, mounting, head-nodding cycles in males, and wing-fanning in females. GC-EAD analysis of female body washes with male antennae revealed seven compounds which elicited antennal responses, four of which are straight-chain alkanes (C_23_, C_25_, C_26_, and C_27_). The identities of these alkanes were confirmed by matching the retention times, mass spectra, and male antennal activity to those of commercially obtained chemicals. In olfactometer assays, a blend of the four straight-chain alkanes was attractive to *I. leucospoides* males, and there was no response to blends that lacked any of these four compounds. Female body wash was no more attractive than the four-component blend. The ratios of EAD-active components differ between hydrocarbon profiles from males and females.

**Conclusion:**

This study is the first investigation of cuticular hydrocarbons in the family Ibaliidae. It provides evidence that the ubiquitous alkanes (C_23_, C_25_, C_26_, and C_27_) in sex-specific ratios attract *I. leucospoides* males.

## Introduction

The parasitic wasp, *Ibalia leucospoides* (Hochenwarth) (Hymenoptera: Ibaliidae), is a solitary endoparasitoid native to the Northern Hemisphere (Hurley et al., 2020). This parasitoid has been extensively employed in biological control programs against the invasive Eurasian woodwasp, *Sirex noctilio* Fabricius (Hymenoptera: Siricidae) in the Southern Hemisphere (Taylor, 1980; Fischbein & Corley, 2015). *S. noctilio* attacks a broad range of *Pinus* species (Carnegie et al., 2006), causes severe damage and mortality in pine plantations, and has been successfully established in the Southern Hemisphere (Hurley, Slippers & Wingfield, 2007; Slippers, Hurley & Wingfield, 2015). As a management plan for the control of *S. noctilio*, multiple exotic biological agents from disparate geographic locations were introduced in various countries of Southern Hemisphere, of which only five parasitoids, including *I. leucospoides*, became established after numerous introductions (Cameron, 2012; Hurley et al., 2020).

The success of *I. leucospoides* as an effective natural enemy of *S. noctilio* is attributed to its high survival and dispersal capacity, emergence synchronization with *S. noctilio* and high fertility and fecundity (Klasmer & Botto, 2012; Ryan et al., 2012; Foelker et al., 2016). This parasitoid has been investigated in studies addressing biological traits, such as the role of food consumption on patch residence time (Corley, Villacide & Van Nouhuys, 2010), flight (Fischbein et al., 2011), longevity, egg maturation (Fischbein et al., 2012), and host searching decisions (Pietrantuono et al., 2012), as well as host-foraging behavior, including interference competition (Corley, Villacide & Van Nouhuys, 2010; Fernández-Arhex & Corley, 2010), host location (Madden, 1968; Spradbery, 1974; Martínez, Fernandez-Arhex & Corley, 2006; Faal, 2020), patch choice decisions (Fischbein et al., 2012), and functional response (Fernández-Arhex & Corley, 2005). Other important behavioral features of this parasitoid have not been adequately addressed, such as mate searching, courtship, and mating behaviors. For parasitoids, these behaviors are frequently mediated by sex pheromones (Ruther, 2013). Highly volatile sex pheromones can attract mates from long distances, and male-male competition and courtship behaviors commence in response to short-range pheromones with lower volatility (Ayasse, Paxton & Tengö, 2001). Parasitoid courtship is often defined as a series of behaviors such as tapping antennae on a substrate, antennal grooming, trail-following, body or abdomen vibration, wing fanning, and mounting (Ayasse, Paxton & Tengö, 2001). The study of courtship and mating behaviors as well as sex pheromones in parasitoids is important because the success of an exotic parasitoid in a biological control program stems from its ability to find mates and increase the female sex ratio to ensure both insect reproduction and population establishment (Hopper & Roush, 1993). They may also be a powerful tool for assessing the establishment and spread of introduced parasitoids.

Within the parasitic Hymenoptera, the existence of sex pheromones has been documented for several dozen families (Ayasse, Paxton & Tengö, 2001; Keeling, Plettner & Slessor, 2004; Boulton, Collins & Shuker, 2015). Most are female-produced sex pheromones including those identified in Braconidae (Swedenborg & Jones, 1992a; Swedenborg & Jones, 1992b; Syvertsen et al., 1995), Ichneumonidae (Eller et al., 1984; Dalen et al., 2015; Marepally & Benarjee, 2016), Figitidae (Weiss et al., 2013), Aphelinidae (Fauvergue, Hopper & Antolin, 1995; Bernal & Luck, 2007), Eurytomidae (Krokos, Konstantopoulou & Mazomenos, 2001), Pteromalidae (Sullivan, 2002; Steiner, Hermann & Ruther, 2006; Ruther, Döring & Steiner, 2011), Chalcididae (Roscoe et al., 2016), and Platygasteridae (Salerno et al., 2012). Several male-produced sex pheromones of parasitic Hymenoptera have been identified; these are from the Pteromalidae (*e.g.*, *Nasonia vitripennis* (Walker)*,* (Ruther et al., 2007) and Eulophidae (*e.g.*, *Melittobia digitata* Dahms, Cônsoli et al., 2002).

The objectives of this study were to observe and describe the sequence of courtship behaviors and to identify semiochemicals that are involved in attraction of this parasitoid to conspecifics.

## Methods

### Insect collection

*Pinus sylvestris* Linnaeus and *P. resinosa* Aiton (Pinaceae) naturally infested with *S. noctilio* were collected at two sites in New York State from January 2015 to April 2019. The locations were Chimney Bluffs State Park (43°16′50″N, 76°54′48″W) in Wolcott, NY, USA and the Pack Demonstration Forest (43°32′58″N, 73°49′12″W) in Chestertown, NY, USA. Fieldwork was approved by the New York State Office of Parks, Recreation, and Historic Preservation (application numbers: 2015-CB-001, 2017-CB-001, 2018-CHB-001). Trees were felled and cut into 1 meter bolts and transported to the laboratory at the SUNY-College of Environmental Science and Forestry in Syracuse, NY, USA. The bolt ends were coated with wax (Gulf Wax, Royal Oak Enterprises, LLC, Roswell, GA) to slow moisture loss, stored at 1−5 °C for two weeks to two months, and every two weeks a subset of logs was transferred to emergence cages at room temperature. After 6-8 weeks adult *I. leucospoides* began emerging and were collected daily and individually stored in 59 ml plastic mini-cups (Wal-Mart Stores, Inc., Bentonville, AR) at 5 °C. We did not observe mating in the emergence cages in the entire time of collections, so we assumed collected wasps were not mated (Fischbein et al., 2012). In addition, we collected and dissected newly emerged females and males and observed them under a compound microscope at 1000 × to assess the presence of spermatozoa in the spermathecae and testes (as a positive control). Spermatozoa were clearly visible in the testes but no spermatozoa were observed in the dissected spermathecae (*n* = 16 females, 10 males).

### Mating behaviors

Courtship behavior of *I. leucospoides* adults was observed in a wire mesh observation cage (0.5 mm mesh, 75 cm length ×10 cm diameter). The observation cage was positioned horizontally on a table with a two-bulb white fluorescent strip light (40 W) centered 75 cm above it. Wasps were taken out of cold storage and held at room temperature (25 ± 3 °C) for at least one hour before starting observations. A male and a female were released at opposite ends and observed for 15 min. Any wasp that did not move or interact with other wasp within 15 min was removed and replaced. All courtship observations (*n* = 14) were conducted at 25 ± 3 °C between the hours of 09:00 and 12:00 and were recorded using an iPhone SE device. All wasps were presumably virgin, and each individual wasp was used only once. The ages of males and females were between 1 and 20 and 1 and 10 days post-emergence, respectively. At the end of each day, wasps were fed only with water (reverse osmosis [RO] water) (Pietrantuono et al., 2012), then refrigerated for other experiments, including olfactometer assays.

### Cuticular hydrocarbons extracts

*Ibalia leucospoides* cuticular hydrocarbons were obtained individually from 1- to 5-day post-emergence virgin adults (males and females) by immersing the whole body of a live wasp into 2 ml of dichloromethane (DCM) (99.9%, OmniSolv, Billerica, USA) for 2 min. Each individual was immersed three times (Böröczky et al., 2009) and the extracts from each insect were combined, concentrated to 200 µl under a gentle stream of nitrogen, and stored at −60 °C until further analysis. A total of 24 extracts were prepared for each sex.

### Body size measurement

The body size of *Ibalia* males is smaller than that of females, so the ratio of male/female body size was applied to the quantities of cuticular components in males as a correction factor for size when the quantities of cuticular components of males was compared to those of females.

To measure the body sizes, measurements were carried out with extracted individuals using a caliper accurate to 0.1 mm. Body surface area of both males (*n* = 32) and females (*n* = 24) was estimated based on the length, width, and height of the thorax (including propodeum but minus wings and legs) and abdomen. The surface areas of the thoraxes and abdomens were calculated as prolate ellipsoids (Wang & Messing, 2004; Macias-Ordonez & Draud, 2005; Wang et al., 2016).

### Identification of EAD-active components

The cuticular hydrocarbon extracts were analyzed with a Gas Chromatograph (GC) (Hewlett-Packard, HP 5890 Series, Sunnyvale, CA, USA) with a flame ionization detector (FID) and a custom electroantennographic detection system. Analytical conditions were as follows: splitless mode, injector temperature 239 °C, detector temperature 240 °C, a non-polar HP5-MS column 30 m × 0.25 mm ID × 0.25 µm film thickness (Agilent Technologies, Santa Clara, CA, USA), and nitrogen carrier gas. The temperature program was 39 °C for 1 min, increased 10 °C/min to 240 °C, and held for 40 min. The GC was coupled with an electroantennographic detection (EAD) system and Clarity Lite integrator (Data Apex Ltd., Czech Republic). The column effluent was split 1:1 in the oven using a Y-splitter (Supelco, Bellafonte, PA, USA); the EAD side had nitrogen make-up gas added (8 ml/min) through a second Y-splitter. Both the FID and the heated EAD port were 280 °C. The EAD half of the effluent was introduced into a cooled humidified air stream (1 L/min) directed toward the antennal mount. Whole head preparations were made of individual insects by excising the head and positioning the antennae between two gold wire electrodes immersed in saline-filled (140 mmol NaCl, 10 mmol KCl, 6 mmol CaCl_2_, 2 mmol MgCl_2_) wells in an acrylic holder. The EAD signal was amplified (10×) with a custom-built high input impedance DC amplifier and signals from both the FID and the antenna were collected simultaneously and digitized with the Clarity Lite integrator and Clarity Lite software (version 7.1.0.151-2016; DataApex Ltd., Czech Republic). The electrophysiological activity of samples and the identified synthetic compounds were confirmed on GC–EAD for both males and females.

The cuticular hydrocarbon extracts were also analyzed on a gas chromatograph (Model 7890A; Agilent Technologies, Inc., Santa Clara, CA, USA) equipped with a mass spectrometric detector (Model 5975c; EI mode, 70 eV with a scanning range of 30.0–550.0 m/z; Agilent Technologies, Inc., Santa Clara, CA, USA) and a non-polar capillary column, HP5-MS (30 m × 0.25 mm ID × 0.25 µm film thickness; Agilent Technologies, Inc., Santa Clara, CA, USA). The carrier gas was helium at a constant flow rate of 1 ml/min. The EAD-active components were tentatively identified by comparison of mass spectra from National Institute of Standards and Technology (NIST) mass spectra (MS), Kovats indices (KI) were calculated relative to straight chain alkanes (Kováts, 1965), and confirmed by comparison with the KI, the retention times and electron impact mass spectra of available compounds. Female and male equivalents for each EAD-active compound were determined by GC-MS quantification using authentic samples of each EAD-active component as external standards to create calibration curves. Components 1 and 2 were quantified using the closest alkane standards (pentacosane and hexacosane) as external standards.

### Synthesis of (6*Z*,9*Z*)-heptadeca-6,9-diene

Barton decarboxylation / bromination was used to synthesize this compound from linoleic acid (Loreau et al., 2000) analogous to (3*Z*,6*Z*,9*Z*)-heptadeca-3,6,9-triene synthesis from linolenic acid adapted from a previously published study of blueberry spanworm (*Itame argillacearia*, (Lepidoptera: Geometridae)). (De Silva et al., 2013) (See Scheme 1 and details in Supplemental Information).

### Chemicals

Chemical standards of tricosane (99%) and pentacosane (99%) were purchased from Sigma-Aldrich Inc. (St. Louis, MO, USA) and BeanTown Chemical (Hudson, NH, USA), respectively. Hexacosane (99%) and heptacosane (99%) were obtained from Alfa Aesar (Ward Hill, MA, USA). (6*Z*,9*Z*)-heptadeca-6,9-diene was synthesized as described above.

### Two-choice olfactometer assays

A Y-shaped glass olfactometer was used to determine male responses to pairs of stimulus blends (Table 1). The olfactometer consisted of a Y-shaped glass olfactometer (6 cm i.d.) with a 26 cm main stem and two 21 cm arms and a 70° angle between the arms. The olfactometer was positioned horizontally on a table with a 500 W halogen lamp centered 50 cm above the Y-tube. Charcoal-filtered air was passed through two Erlenmeyer flasks containing 100 ml water, connected to plastic tubing (1 m length), and attached to polytetrafluoroethylene (PTFE) tubing (10 cm length) at the ends of each of the choice arms. The air flow rate in each of the choice arms was 500 ml/min, and at the base the rate was 1000 ml/min. Air was removed from the base by vacuum. Cardboard (100 cm length × 60 cm width) surrounded the olfactometer to eliminate visual cues. A piece of filter paper (5 cm × 0.5 cm) was loaded with 5 µl of extract or solution and was placed at the end of a choice arm. For each trial, a male was released at the base of the main arm and was allowed 6 min to respond to an odor source by reaching the end of a choice arm. The positions of odor sources and the olfactometer arms were alternated after every trial to control for potential positional bias. The Y-tubes were washed between trials after every responsive insect with unscented detergent (Alconox, Jersey City, NJ, USA) and hot water, rinsed with RO water, and baked at 120 °C for at least 1 h. At the end of each day, the glassware was washed and baked overnight. All bioassays were carried out between 07:00 and 18:00 at 25 ± 3 °C, and each male was used no more than once per day. Males were re-used in different olfactometer experiments using different stimuli and none was reused for the same pair of stimuli, *i.e.,* in the same experiment. A positive response was recorded when a male reached the end of the arm containing a stimulus, while a negative response was recorded for those that reached the end of the arm containing the control within 6 min. All males were 1–14 day old virgins, and were provided with RO water for one day before being used in assays (Pietrantuono et al., 2012). Females were not used in the olfacometer assays because only male attraction to females was observed in our study of mating behavior.

**Table 1:** Components of each blend used in behavioral assays for male *Ibalia leucospoides*.

Component	Amount (ng)^a^	Blends					
		1	2	3	4	5	6
(Z,Z) 6,9, heptadecadiene	1.4	×					
Tricosane	3.25	×	×		×	×	×
Pentacosane	275	×	×	×		×	×
Hexacosane	1000	×	×	×	×		×
Heptacosane	1450	×	×	×	×	×	

**Notes.**

a Each blend used in each olfactometer assay was equal to 0.25 Female Equivalent.

The following assays were conducted: (1) female body wash *vs.* DCM, (2) male body wash *vs.* DCM, (3) a blend of the five authentic compounds (Blend 1, commercially obtained or synthesized) (Table 1) *vs.* DCM, (4) Blend 1 *vs.* a blend of the four commercially available alkanes (Blend 2), (5) a series of subtractive assays in which one component was removed from Blend 1 at a time and the remaining blend was assayed against DCM, (6) Blend 2 *vs.* female body wash. The component ratios in the blends approximated those in extracts obtained from individual females and measured with GC-MS (Table 1).

### Statistical analysis

The equality of variance and normality of body size measurements (surface area) were confirmed using the Kolmogorov–Smirnov test. The Student’s two-tailed t test was used to compare the surface area measurements for both males and females. The amount of each EAD-active component in individual females was multiplied by 0.75 to adjust for sexual dimorphism in surface area. The Student’s two-tailed t test was used to compare the quantity of each EAD-active component between males and females. *P*-values were compared with the Bonferroni corrected alpha level (0.05/7 = 0.0071). For each trial in Y-tube assays, the numbers of wasps that responded to stimuli were compared with a binomial test with expected 1:1 ratio at a probability of 5%. All statistical analyses were conducted in SAS 9.4.

## Results

### Mating behaviors

A consistent sequence of courtship behaviors was observed. Once a male was released into an arena, it groomed its antennae with its mouthparts and front legs, and progressively walked toward the female. Once it encountered the female, the male occasionally bounced its abdomen vertically, approached from the rear or side, and initiated rhythmical lateral movements while holding its antennae upright. Meanwhile, the female slowly walked in the opposite direction and slightly vibrated its wings (wing fanning). The female then groomed its antennae with its front legs, occasionally bounced its abdomen vertically, and waited for the male to arrive. The male climbed onto the female (mounted) from the side while making antennal contact, and aligned its body with its head above that of the female. Once the male mounted the female, it initiated head-nodding behavior, starting with multiple fast strokes with its antennae against the female antennae, followed by slow upward sweeping movements. All strokes and sweeping movements occurred with one male antenna against one female antenna, alternating antennae at the end of each head-nodding cycle. The duration of each cycle was 3.5 ±0.4 s, and each male performed 17.7 ± 0.6 head-nodding cycles for a total of 62 ± 8.1 s (*n* = 14). Females held their antennae upright with no movement during the male head-nodding cycles. Two types of responses were observed in females: (1) in most cases the female ignored the male, particularly smaller and older males, and did not allow the male to mount, or (2) the male immediately mounted and initiated the head-nodding cycle, but the female was not sexually receptive, and kicked the male off with her back legs after multiple kicks. Meanwhile, males exhibited a short bout of wing fanning immediately following each rejection. This was the only instance in which males exhibited wing fanning. We observed no successful copulations (*n* = 14), although the majority of males tried to mount the female and then repeated the cycle after rejection by the female. Our observations showed that the majority of males, regardless of size or age, were attracted to females, although females only allowed males of similar or larger size to mount.

### Body size measurements

There was no significant difference between thoracic surface area measurements between males and females (Student two-tailed t test, *P* > 0.05, *t* = 0.68) (Table 2). The abdominal surface area and the total surface area measurements of *I. leucospoides* females were significantly greater than those of males (Student two-tailed t test, *P* < 0.05, *t* = 3.75 for abdomen, and *P* < 0.05, *t* = 2.48) (Table 2).

**Table 2:** Body sizes of *Ibalia leucospoides* males (*n* = 32) and females (*n* = 24).

Sex	Body size								
	Thorax^1^				Abdomen				Total Surface Area (mm^2^)
	Length (mm)	Width (mm)	Depth (mm)	Surface Area (mm^2^)^2^	Length (mm)	Width (mm)	Depth (mm)	Surface Area (mm^2^)	
Female	2.98 ± 0.15	1.60 ± 0.09	1.71 ± 0.08	12.9 ± 1.18 a^3^	5.77 ± 0.22	0.1 ± 0.0	2.31 ± 0.12	20.4 ± 1.64 a	34.61 ± 2.77 a
Male	2.83 ± 0.08	1.54 ± 0.06	1.66 ± 0.05	12.6 ± 0.66 a	4.72 ± 0.14	0.1 ± 0.0	1.88 ± 0.04	14.3 ± 0.66 b	26.9 ± 1.26 b

**Notes.**

1 Includes propodeum.

2 The relative body surface area was calculated for both thorax and abdomen, which were both assumed to be a prolate ellipsoid (Wang & Messing, 2004; Macias-Ordonez & Draud, 2005; Wang et al., 2016).

3 Values (mean ± S.E.) followed by the same letter within each column were not significantly different (Student two-tailed t test, *P* > 0.05).

### Identification of EAD-active components

**Figure 1:**
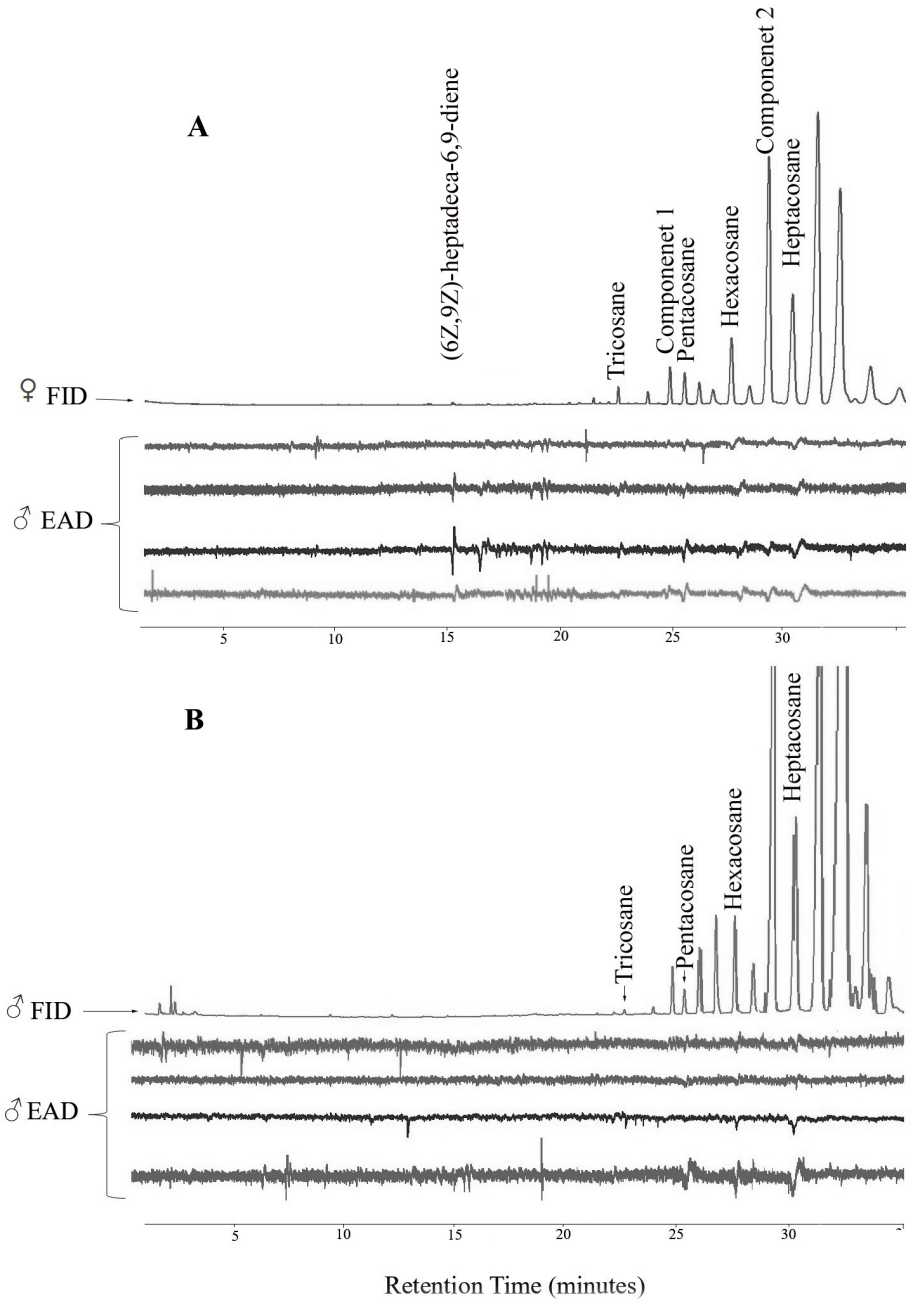
Representative coupled gas chromatography-electroantennogram detection analysis of body wash extracts of *Ibalia leucospoides* female (A) and male (B), using antennae from *I. leucospoides* males.

**Figure 2:**
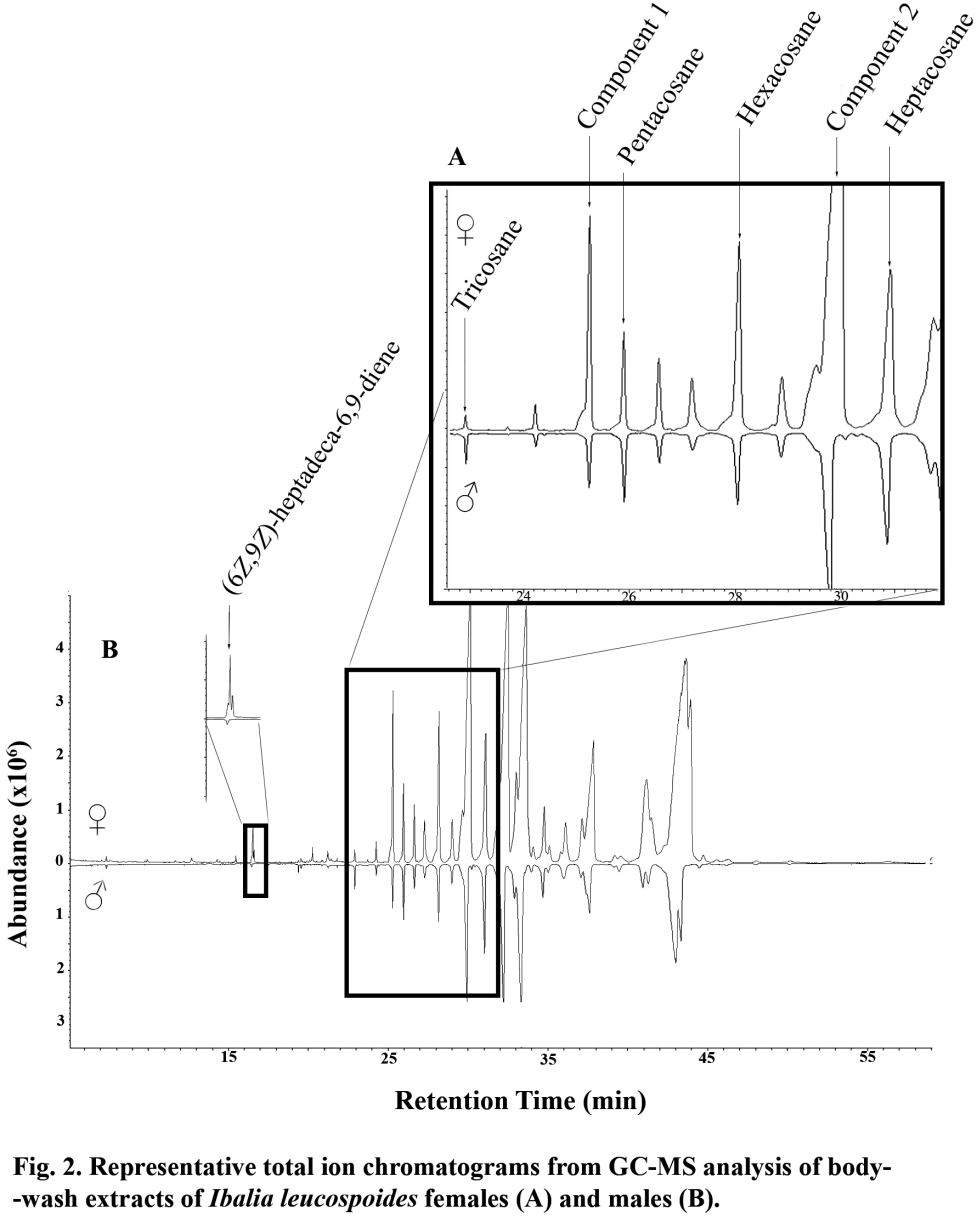
Representative total ion chromatograms from GC-MS analysis of body wash extracts of *Ibalia leucospoides* females (A) and males (B).

In GC-EAD analyses of female body washes, antennae of males repeatedly responded to seven components present in all extracts (Table 3, Fig. 1). In GC-MS analyses of female body washes, the retention time and mass spectra of five of the components matched those of (6*Z*,9*Z*)-heptadeca-6,9-diene (supplemental information) tricosane, pentacosane, hexacosane, and heptacosane (Fig. 2). Although the retention time, EI-mass spectra and KI of the synthetic diene support this structure, the identification of this compound remains tentative. Further microchemical analyses (*e.g.*, epoxidation) followed by GC/MS analysis, were not successful in producing structural information because of high background. The mass spectra of two additional EAD-active components are indicative of the branch point; 2-methylalkanes give highly characteristic EI-mass spectra showing enhanced M-15 and M-43 (loss of an isopropyl radical leaving the positive charge on the primary fragment). Authentic compounds were not available. The Kovats indices of them matched with two methylbranched alkanes (2-methyltetracosane and 2-methylhexacosane; components 1 and 2, respectively, see supplemental information). All EAD-active components were present in all male and female body washes, except (6*Z*,9*Z*)-heptadeca-6,9-diene, which was present in all female samples, but found in low quantity in the body washes of only seven of forty males. The amount of each EAD-active component was compared between males and females after correcting for the sexual dimorphism in body surface area. The statistical analysis showed that the amounts of C_25_, C_26_, (6*Z*,9*Z*)-heptadeca-6,9-diene, and component 1 were significantly different between males and females (*P* < 0.05).

**Table 3:** Electrophysiologically active components of female *Ibalia leucospoides* body washes.

Component	Kovat’s index^1^ (reference)	Females (*n* = 23)	Males (*n* = 37)	t (P)	Ratios Male/Female
		Amount (*μ*g) ± S.E.^2^			
(6*Z*,9*Z*)-heptadeca-6,9-diene	1670 (1662^3^)	0.03 ± 0.0 a^7^	0.00 ± 0.0 b	2.93 (<0.001)	0.04
Tricosane	2300 (2300^4^)	0.06 ± 0.01 a	0.04 ± 0.0 a	0.99 (0.32)	0.66
Pentacosane	2500 (2500^4^)	5.4 ± 0.6 a	2.5 ± 0.2 b	3.95 (<0.001)	0.46
Hexacosane	2600 (2600^4^)	20 ± 2.3 a	11.3 ± 0.8 b	4.42 (<0.001)	0.56
Heptacosane	2700 (2700^4^)	29 ± 3.4 a	26.7 ± 2.5 a	0.08 (0.94)	0.92
Component 1^8^	2460 (2465^5^)	9.2 ± 1.1 a	4.4 ± 0.4 b	3.92 (<0.001)	0.48
Component 2	2670 (2665^6^)	78.1 ± 8.4 a	56 ± 5 a	0.85 (0.39)	0.72

**Notes.**

1 The calculated Kovat’s indices of compounds eluted on a non-polar HP5-MS column.

2 Amount of each component and standard errors correspond to those found in individual body wash extracts.

3 Tsai et al. (2019).

4 Zgheib et al. (2020).

5 Zaikin & Borisov (2002).

6 Hedlund et al. (1996).

7 Values (mean ± S.E.) were compared after adjusting for sexual dimorphism in surface area. Values with the same letter within each row were not significantly different (Student two-tailed t test, *P* > 0.05).

8 Components 1 and 2 were quantified using the authentic samples of pentacosane and hexacosane as external standards.

### Two-choice olfactometer assays

**Figure 3:**
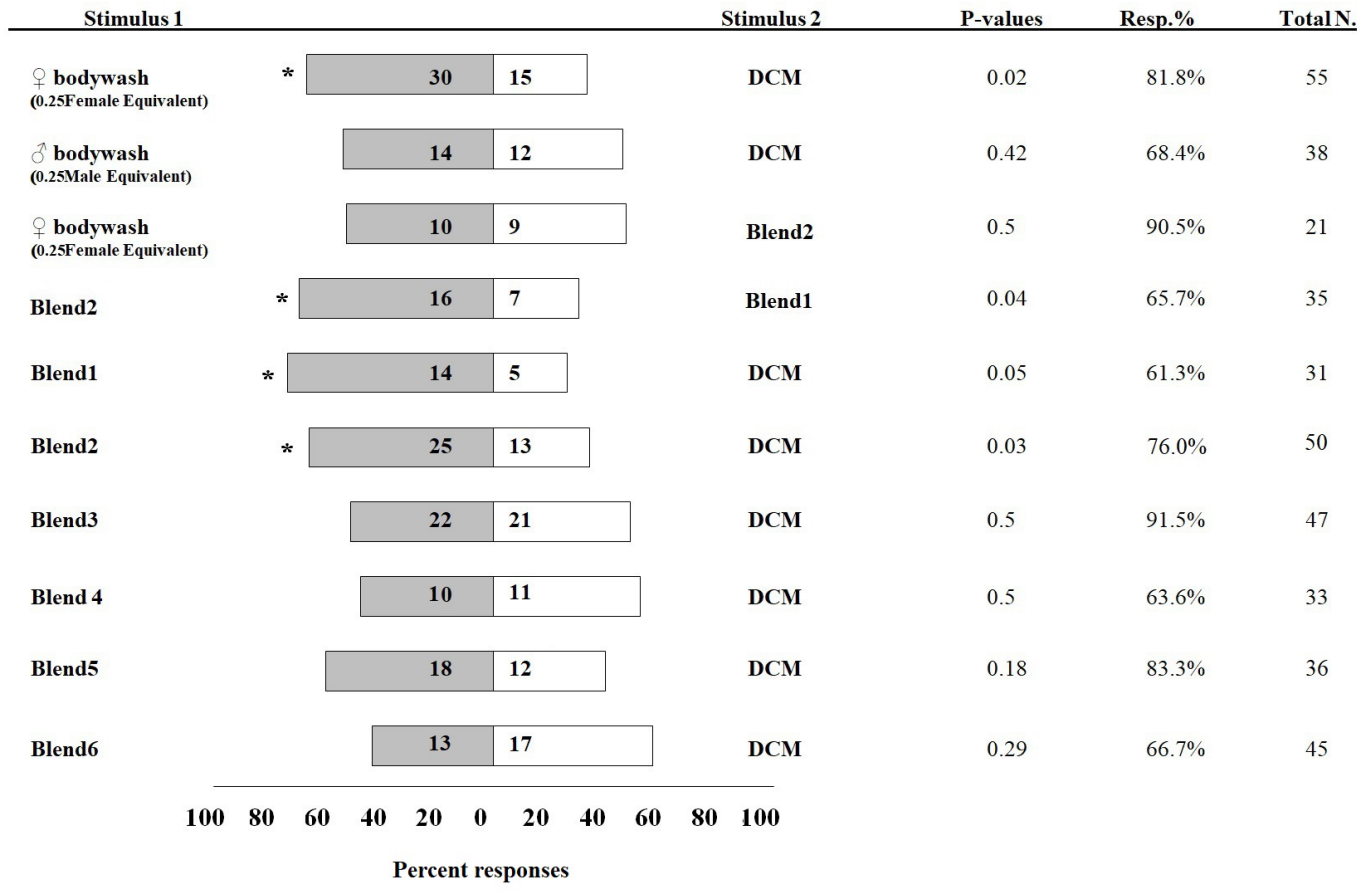
Percentage of *Ibalia leucospoides* male responses to different stimuli in olfactometer assays. The authentic blends were prepared at the same concentrations and ratios found in body wash extracts of individual *I. leucospoides* females. Table 1 provides the composition of each blend. The number of insects that responded to the stimuli are noted next to the corresponding bar. The *P*-value, percent responding (Resp.%), and total number of wasps for each trial is presented adjacent to the corresponding bar. The significant differences at *α* =0.05 are indicated by asterisks.

In olfactometer assays, 30 *I. leucospoides* males were attracted to female body washes, compared to 15 males that responded to DCM alone (*P* < 0.05) (Fig. 3). *I. leucospoides* males were not attracted to male body washes. The five-component blend (Blend 1) was attractive to *I. leucospoides* males (*P* = 0.05). Similarly, 25 males responded to odors of the four-component Blend 2, compared to only 13 that responded to DCM (P = 0.03). Blend 2 was more attractive than Blend 1 to *I. leucospoides* males (*P* = 0.04). Male *I. leucospoides* were not attracted to other blends in which individual components were successively removed from Blend 2 (Fig. 3). When the female body wash was tested for male attractiveness against the four-component blend (Blend 2), Blend 2 was no more attractive to males than the female body wash (*P* = 0.5).

## Discussion

Our study is the first description of the courtship behavior and identification of attractive components in the cuticular hydrocarbons of *I. leucospides*. Our observations of the courtship behavior of *I. leucospoides* revealed a consistent sequence of behaviors, including rhythmic lateral movements, approaching from the rear or side, mounting, head-nodding cycles in males, and wing-fanning, antennal grooming, and abdominal bouncing in females. These observations are consistent with previously reported courtship behavior sequences in other hymenopteran parasitoids (Vinson, 1972; Sullivan, 2002; Roscoe et al., 2016).

Rhythmic lateral movements were only observed in males when the male was in close proximity to a female. Similar behavior was not observed in Y-tube assays when males were exposed to both the female body wash extract and the multi-component blend. It could also be a result of visual cues. Several explanations have been proposed for these rhythmic lateral movements in other parasitic Hymenoptera, including enhancing volatile detection and mate recognition (Leonard & Ringo, 1978; Roscoe et al., 2016), or observing immobile females (Roscoe et al., 2016). In *I. leucospoides*, this behavior may be a visual cue that plays a role in mate recognition. The male head-nodding cycle has also been observed in pteromalids including *Roptrocerus xylophagorum* (Ratzeburg) (Sullivan, 2002) and several *Nasonia* species (reviewed by Mair & Ruther, 2019). However, the length and number of cycles were different for each species tested (Mair & Ruther, 2019). Some males may provoke receptiveness in females by releasing a pheromone from their mouthparts or antennal exocrine glands during antennal stroking (Van den Assem, Jachmann & Simbolotti, 1980; Isidoro, Romani & Bin, 2001).

The wing fanning of *I. leucospoides* females may assist males in orienting toward mates. Similar behavior was observed in the parasitoid *Campoletis sonorensis* (Cameron) (Hymenoptera: Ichneumonidae) (Vinson, 1972). Wing fanning may indicate recognition of a conspecific adult (Sullivan, 2002; Danci et al., 2006) or be an indicator of male quality for females (Benelli et al., 2014).

We did not observe successful copulation, perhaps because females were mated in the emergence cage before the daily collection, or the observations were made outside the time window of normal daily mating activity. To our knowledge, there is no record of the mating activity of *I. leucospoides* in the literature. Spradbery (1970) reported successful mating behavior of *I. drewseni* Borries in an observation cage. The duration of copulation of *I. drewseni* was 0.3 to 2.9 min (*n* = 10) and both sexes attempted to mate multiple times (Spradbery, 1970). In order to understand the mating sequence of *I. leucospoides*, additional studies are required to analyze visual or chemical stimuli, or both, under controlled conditions.

Our results provide evidence of the attraction of *I. leucospoides* males to the cuticular hydrocarbons of female conspecifics. Seven EAD-active components were identified: four long-chain alkanes with 23, 25, 26, and 27 carbons, (6*Z*,9*Z*)-heptadeca-6,9-diene, 2-methyltetracosane, and 2-methylhexacosane. Interestingly, male body wash extracts were not attractive to males in olfactometer assays, although all identified alkanes exist in male body washes. It is possible that the sex-specific ratios of the alkanes (C_23_, C_25_, C_26_, and C_27_) play an important role in the attraction of males to female, but not male, body wash extracts. In *I. leucospoides*, all EAD-active components exist in both sexes but in different ratios, except (6*Z*,9*Z*)-heptadeca-6,9-diene, which was consistently present and in significantly greater relative quantities in female body washes compared to male body washes in which it occurred in fewer than 20% of the individuals sampled.

In olfactometer assays, a four-component blend of long-chain alkanes with 23, 25, 26, and 27 carbons was attractive to *I. leucospoides* males, which did not respond to the blend in the absence of any single component. A combination of all four straight-chain alkanes was needed to attract males in olfactometer assays. Hydrocarbons and other lipids in this size range are commonly used for short-range pheromones, which facilitate mate recognition or courtship coordination (Keeling, Plettner & Slessor, 2004; Johansson & Jones, 2007; Kather & Martin, 2015). For instance, male courtship sequences in *N. vitripennis* are mediated by a fraction of female cuticular hydrocarbons with chain lengths between 25 and 37 carbons (Steiner, Hermann & Ruther, 2006). We do not know the active range of the four-component blend for *I. leucospoides* males. However, the chain lengths and therefore the volatility of the identified alkanes in cuticular hydrocarbons of *I. leucospoides* females suggest short-range activity (Syvertsen et al., 1995; Steiner, Hermann & Ruther, 2006). (6*Z*,9*Z*)-heptadeca-6,9-diene was present in all female body wash extracts, but only in seven male extracts, and at a significantly lower amounts than in female body washes. This evidence initially suggested that (6*Z*,9*Z*)-heptadeca-6,9-diene might be an important component of the *I. leucospoides* sex pheromone. However, in two-choice olfactometer assays, Blend 2 was more attractive than Blend 1. We did not test the activity of (6*Z*,9*Z*)-heptadeca-6,9-diene alone, and future assays are needed to determine its activity and function. The same compound, (6Z,9Z)-heptadeca-6,9-diene, has been identified as a sex pheromone of geometrid and noctuid moths (Ando et al., 1995), as well as an alarm pheromone of the Astigmatid mite, *Tortonia* sp., which feeds on pollen masses of the megachilid bee, *Osmia cornifrons* (Kuwahara et al., 1995a; Kuwahara et al., 1995b). Contact behaviors (antennation and mounting) in *Cardiochiles nigriceps* (Viereck) (Hymenoptera: Braconidae) are mediated by the (7Z,13Z)-7, 13-heptacosadiene (Syvertsen et al., 1995).

Tentative identification indicated that our components 1 and 2 are methylbranched alkanes (2-methyltetracosane and 2-methylhexacosane, respectively). Methylbranched alkanes and alkenes possess additional structural features for species-specific discrimination over straight-chain alkanes in the pteromalid *Lariophagus distinguendus* (Kühbandner et al., 2013). Blends of long chain methylbranched alkanes have been identified as contact sex pheromones for two parasitoids: a blend of 3-methylnonacosane and 3-methylhentriacontane for the pteromalid parasitoid *Dibrachys cavus* (Walker) (Ruther, Döring & Steiner, 2011) and a blend of 5-methylheptacosane and 5,17-dimethylheptacosane for *Ooencyrtus kuvanae* (Howard) (Hymenoptera: Encyrtidae) (Ablard et al., 2012). A female-specific blend of methyl 6-methylsalicylate, fatty alcohol acetates, and cuticular hydrocarbons elicited male courtship behavior in *Asobara tabida* (Nees) (Hymenoptera: Braconidae)*,* a larval parasitoid of *Drosophila* sp. (Stökl, Dandekar & Ruther, 2014). For current study, the methylbranched alkanes were not commercially available, but more importantly, the four alkane blend elicited the same level of attraction from males as the female body washes indicating that all of the activity of the female body washes was attributable to the alkanes. However, the activity of other EAD-active components remains to be determined.

Numerous sex pheromones with different ranges of activity have been identified for hymenopteran parasitoids, including short range sex pheromones with low volatility (Sullivan, 2002; Steiner, Hermann & Ruther, 2006; Salerno et al., 2012) and long range sex pheromones with high volatility (Eller et al., 1984; Swedenborg & Jones, 1992a; Swedenborg & Jones, 1992b). Also, the behavioral observations of several male parasitoids have confirmed the existence of substrate-borne sex pheromones for *Ascogaster reticulatus* Watanabe (Hymenoptera: Braconidae) (Kamano et al., 1989; Kainoh et al., 1991; Kainoh & Oishi, 1993), *Aphelinus asychis* Walker (Hymenoptera: Aphelinidae) (Fauvergue, Hopper & Antolin, 1995), *Trichogramma brassicae* Bezdenko (Hymenoptera: Trichogrammatidae) (Pompanon et al., 1997), and *Glyptapanteles flavicoxis* Ashmead (Hymenoptera: Braconidae) (Danci et al., 2006). In some cases, the majority of the males actively explored areas previously visited by virgin females (Kamano et al., 1989; Fauvergue, Hopper & Antolin, 1995; Pompanon et al., 1997; Danci et al., 2006).

Analyses of cuticular hydrocarbon profiles of parasitic wasps have shown them to be species and sex-specific (Howard & Blomquist, 2005; Ruther, 2013). In most cases, one or more distinctive hydrocarbons occur in only one sex (Darrouzet et al., 2010; Raychoudhury et al., 2010; Salerno et al., 2012; Buellesbach et al., 2013; Weiss, Ruther & Stökl, 2015; Bien et al., 2019; Ruther et al., 2019), while in some cases, the same hydrocarbons occur in both sexes but in different proportions (Howard, 1998; Howard, Pérez-Lachaud & Lachaud, 2001), or in similar proportions (Howard, 1992; Howard & Liang, 1993; Howard & Pérez-Lachaud, 2002; Howard & Baker, 2003). The majority of the documented cuticular hydrocarbons in parasitoid wasps may act as short-range pheromones but this has yet to be explicitly demonstrated. Interestingly, the orchid *Ophrys* produces several straight-chain saturated and unsaturated hydrocarbons with 21-29 carbons in component ratios that mimic those of a female bee pollinator, *Andrena nigroaenea* and both orchid and female produced blends attract males (Schiestl et al., 1999).

Our results demonstrated the attraction of *I. leucospoides* males in a Y-tube olfactometer to a blend of n-alkanes, which is significant because previous studies reported the attraction of males only after contact with females (Sullivan, 2002; Steiner, Hermann & Ruther, 2006; Salerno et al., 2012). A blend of four long chain alkanes (C_23_, C_25_, C_26_, and C_27_) was attractive in behavioral assays. It is interesting that *I. leucospoides* employs ubiquitous compounds to locate a mate, but a specific component ratio of otherwise common compounds may be the key. Because males did not discriminate between the blend of four alkanes and the female body wash, we concluded that the other EAD-active components (the diene and two methylbranched alkanes) are not essential for male attraction in the laboratory. This could potentially change when these compounds are eventually field-tested. An integrated approach using the identified attractive components of the symbiotic fungal volatiles (Faal et al., 2021), sex pheromones of *S. noctilio* (Faal, 2020), and sex pheromones of *I. leucospoides* is likely required for monitoring populations of both *Sirex noctilio* and its parasitoid.

## Supplemental Information

10.7717/peerj.12266/supp-1 Supplemental Information 1 Raw Data for Fig. 3

10.7717/peerj.12266/supp-2 Supplemental Information 2 Raw Data for Table 2

10.7717/peerj.12266/supp-3 Supplemental Information 3 Raw Data for Table 3

10.7717/peerj.12266/supp-4 Supplemental Information 4 Supplementary data

## Additional Information and Declarations

### Field Study Permissions

The following information was supplied relating to field study approvals (i.e., approving body and any reference numbers): Infested trees were collected from Chimney Bluffs State Park, NY. Fieldwork was approved by the York State Office of Parks, Recreation, and Historic Preservation (application numbers: 2015-CB-001, 2017-CB-001, 2018-CHB-001).

### Data Availability

The following information was supplied regarding data availability: The raw data are available as Supplemental Files.
