# Supplementary material for: Courtship behavior and identification of a sex pheromone in Ibalia leucospoides (Hymenoptera: Ibaliidae), a larval parasitoid of Sirex noctilio (Hymenoptera: Siricidae)
Source: PeerJ. 2021 Nov 4;9:e12266. doi: 10.7717/peerj.12266 (PMC8572519; doi:10.7717/peerj.12266)
Supplement: Supplemental Information 4 [file peerj-09-12266-s004.docx]

SUPPLEMENTARY INFORMATION

Synthesis of linoleic acid chloride

A flame dried round-bottomed flask was charged with linoleic acid ((9*Z*, 12*Z*)-9, 12-octadecadienoic acid) (1.1 mL, 3.5 mmol), oxalyl chloride (0.33 mL, 3.9 mmol), anhydrous CH_2_Cl_2_ (8 mL), and *N*, *N*-dimethylformamide (30 µL, 0.39 mmol). The reaction was stirred at reflux for 6 h, then cooled to room temperature, and the solvent was removed *in vacuo*. The resulting linoleic acid chloride ((9*Z*, 12*Z*)-9, 12-octadecadienoyl chloride) was used in the next step without further purification.

Synthesis of (6*Z*, 9*Z*)*-*17-bromo-6, 9-heptadecadiene

A flame dried round-bottomed flask was charged with dimethylaminopyridine (DMAP, 54 mg, 0.44 mmol), 2-mercaptopyridine *N*-oxide sodium salt (725 mg, 4.86 mmol), and bromotrichloromethane (15 mL, 152 mmol), and this mixture was heated to reflux. Then, linoleic acid chloride ((9*Z*, 12*Z*)-9, 12-octadecadienoyl chloride, ~3.5 mmol, from the last step) was dissolved in bromotrichloromethane (10 mL, 101 mmol) and this was added to the refluxing DMAP / 2-mercaptopyridine *N*-oxide sodium salt mixture dropwise. After refluxing for 2 h, the mixture was cooled to room temperature and diethyl ether (40 mL) and saturated sodium chloride solution (20 mL) were added. The layers were separated, and the aqueous layer was further extracted with diethyl ether (2 × 20 mL). The combined organic extractions were washed with water (30 mL), saturated sodium chloride solution (30 mL) and dried (MgSO_4_). Column chromatography on silica gel (hexanes as eluent) yielded (6*Z*, 9*Z*)*-*17-bromo-6, 9-heptadecadiene (460 mg, 1.46 mmol, 42 % over 2 steps) as a colourless, transparent liquid.

Synthesis of (6*Z*, 9*Z*)-6, 9-heptadecadiene

A flame dried round-bottomed flask was charged with (6*Z*, 9*Z*)*-*17-bromo-6, 9-heptadecadiene (460 mg, 1.46 mmol), anhydrous tetrahydrofuran (10 mL) and lithium aluminum hydride (61 mg, 1.6 mmol). The reaction was stirred at reflux for 3 h, then H_2_O (0.3 mL) was carefully added, followed by 15 % NaOH_(aq)_ (0.3 mL). When the grey colour of the lithium aluminum hydride had discharged, then cloudy white suspension was filtered through celite, and eluted with diethyl ether (20 mL). The resulting solution was washed with brine (20 mL) and dried (MgSO_4_). Column chromatography on silica gel (hexanes as eluent) yielded (6*Z*, 9*Z*)-6, 9-heptadecadiene (260 mg, 1.10 mmol, 75 %) as a colourless, transparent liquid, with the following spectral data: ^1^H NMR (CDCl_3_, 400 MHz): δ 5.30-5.41 (m, 4H), 2.78 (t, 2H, *J* = 6.6 Hz), 2.03-2.08 (m, 4H), 1.28-1.39 (m, 16H), 0.89 (t, 3H, *J* = 6.7 Hz), 0.88 (t, 3H, *J* = 6.5 Hz). ^13^C NMR (CDCl_3_, 100 MHz): 130.2, 130.1, 128.0, 127.9, 31.9, 31.6, 29.7, 29.4, 29.3, 29.2, 27.3, 27.2, 25.6, 22.7, 22.6, 14.09, 14.05. LRMS (EI, 70 eV) m/z main peaks: 55 (44 %), 67 (100 %), 81 (88 %), 95 (69 %), 110 (36 %), 124 (20 %), 138 (15 %), 152 (4 %), 166 (3 %), 236 (26 %, M^+^).


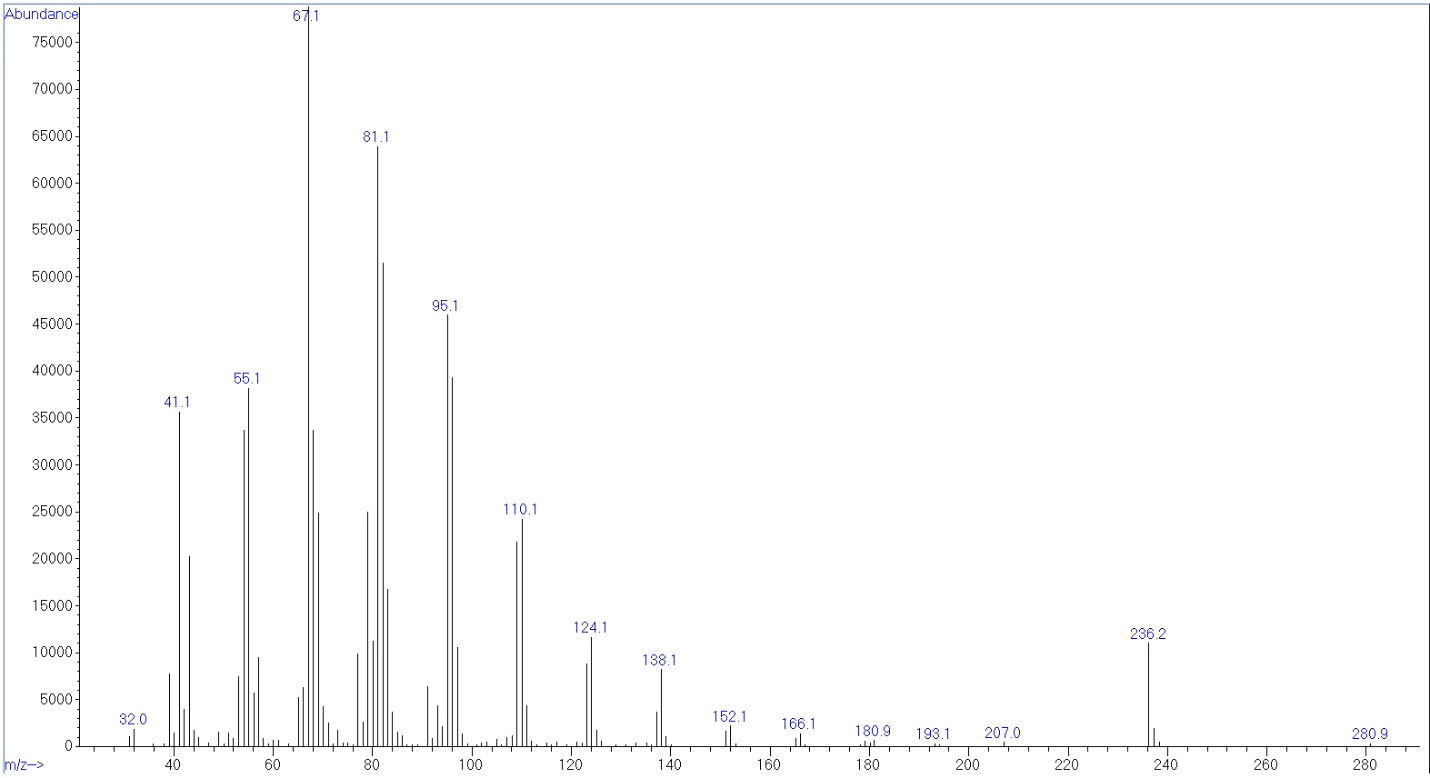
 MS of (3Z,9Z) 6,9, heptadecadiene


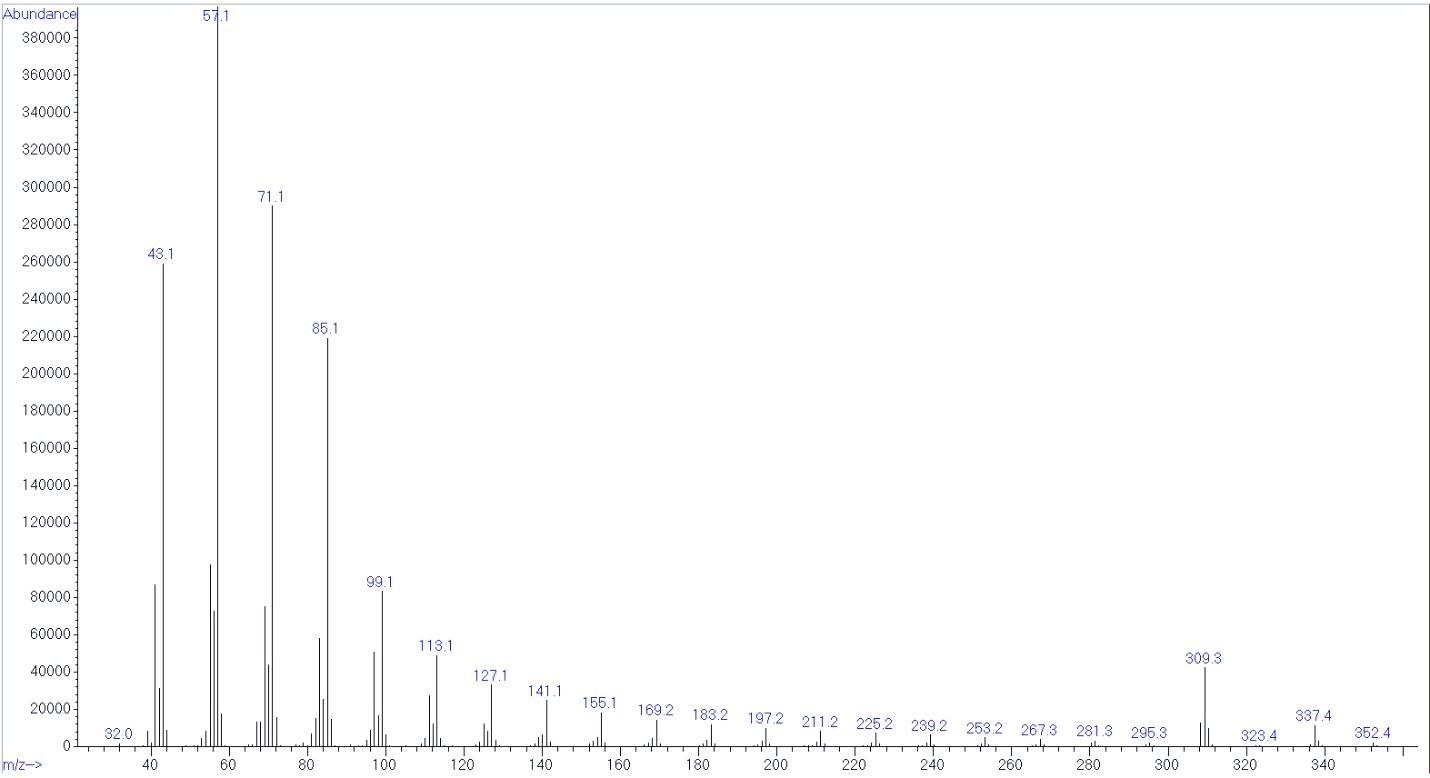
MS of component 1


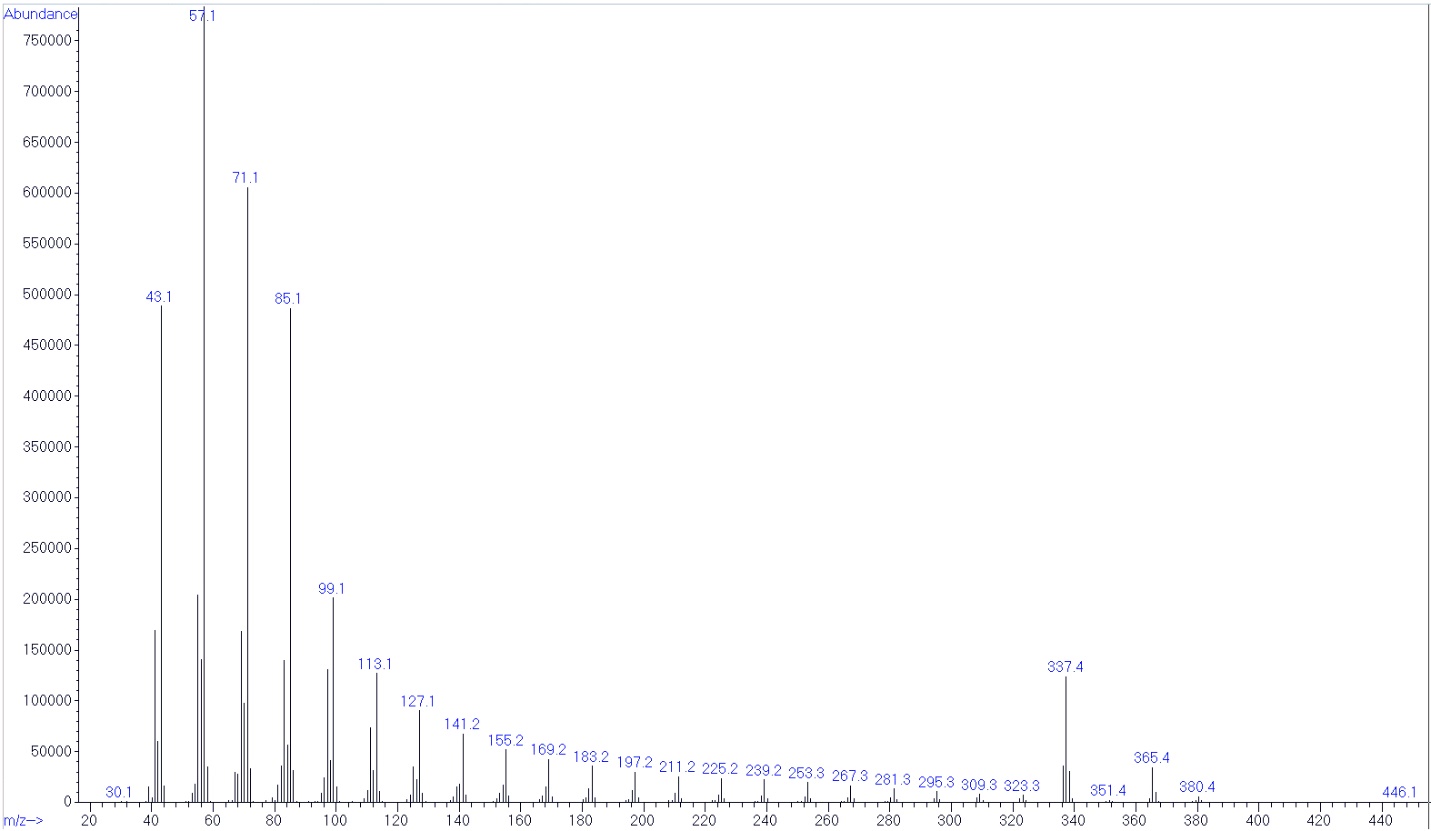
MS of component 2
